# Supplementary material for: Intersections of informal work status, gender and tuberculosis diagnosis: Insights from a qualitative study from an Indian setting
Source: PLoS One. 2023 Jul 27;18(7):e0289137. doi: 10.1371/journal.pone.0289137 (PMC10374003; doi:10.1371/journal.pone.0289137)
Supplement: S1 Table — (DOCX) [file pone.0289137.s001.docx]

**Baseline Data**

| **Sl. No** | **CODE OF THE PATIENT** | **AGE** | **OCCUPATION(NATURE OF WORKING)** | **Pulmonary/Extra pulmonary** | **MDR** | **Co-morbidity** | **Status** |
| --- | --- | --- | --- | --- | --- | --- | --- |
| **1** | WPBG43 | 28 | HOUSE KEEPING | P |  |  |  |
| 2 | SBKG59 | 38 | GARMENTS | EP |  |  |  |
| 3 | WPBG40 | 24 | SALES | EP |  |  | Recurring |
| 4 | WGKG83 | 31 | HOUSE KEEPING | EP |  |  |  |
| 5 | WPMG30 | 39 | HOUSE KEEPING | EP |  |  |  |
| 6 | WPJG01 | 42 | ANGANWADI TEACHER | P |  | DIABETIC |  |
| 7 | WPJG17 | 29 | HOUSE KEEPING | P |  |  |  |
| 8 | WPMG23 | 24 | METRO | P |  |  |  |
| 9 | WPPG32 | 21 | SALES | EP |  |  |  |
| 10 | WGRG95 | 40 | HOSPITAL LONDERY SECTION | P |  |  |  |
| 11 | SBYK052 | 34 | SHOP WORKER | EP |  |  |  |
| 12 | WGSK067 | 33 | ATTENDOR | EP |  |  |  |
| 13 | EKAK044 | 32 | HOUSE KEEPING | EP |  |  |  |
| 14 | WDSK034 | 16 | FACTORY | EP |  |  |  |
| 15 | WPMK020 | 20 | GARMENTS | P |  |  |  |
| 16 | WPMG31 | 42 | AGARBATHI FACTORY | EP |  |  |  |
| 17 | WDSK033 | 30 | WORKED IN SHOPS | EP |  | DIABETES |  |
| 18 | WGGG86 | 23 | SYSTEM WORK | P |  |  |  |
| 19 | EKAG49 | 30 | CARE TAKER | EP |  |  |  |
| 20 | SBYK045 | 37 | HOUSE KEEPING | EP |  |  |  |
| 21 | SBYK050 | 22 | DOMESTIC HELP | P |  |  | Recurring |
| 22 | SBYKO47 | 29 | PRIVATE COMPANY | P |  |  |  |
| 23 | SBYKO59 | 30 | HOUSE KEEPING | EP |  |  |  |
| 24 | SBYK046 | 26 | DOMESTIC HELP | EP |  |  |  |
| 25 | SBYK049 | 35 | TEACHER | EP |  |  |  |
| 26 | SBYK051 | 55 | STREET VENDOR | P |  |  |  |
| 27 | SBYK054 | 31 | RECEPTIONEST | EP |  |  |  |
| 28 | SBKG54 | 31 | PACKING SECTION | P |  |  | Recurring |
| 29 | SBKG52 | 33 | DATA ENTRY | EP |  |  | Recurring |
| 30 | SBKG56 | 27 | CLOTH FACTORY | EP |  |  |  |
| 31 | SBKG61 | 20 | FACTORY | P |  |  |  |
| 32 | SBKG55 | 35 | SILK FACTORY | P |  |  |  |
| 33 | SBVG72 | 28 | FACTORY | EP |  |  |  |
| 34 | SHHG76 | 28 | DATA ENTRY | EP |  |  |  |
| 35 | EAKG10 | 27 | SALOON | P |  |  |  |
| 36 | EKAK009 | 19 | TELECALLER | EP |  |  |  |
| 37 | EKAK007 | 40 | HOUSE KEEPING | EP |  |  |  |
| 38 | EKAK010 | 48 | HOUSE KEEPING | EP |  | DIABETES |  |
| 39 | EKAK031 | 35 | HOUSE KEEPING | EP |  |  |  |
| 40 | EKAK071 | 37 | HOTEL | P |  |  |  |
| 41 | EKAK032 | 40 | DOMESTIC HELP | P |  |  |  |
| 42 | WGGG92 | 23 | ORIAN MALL | EP |  |  |  |
| 43 | WGGK072 | 20 | OFFICE | P |  |  | Recurring |
| 44 | WGRG93 | 20 | SALES | EP |  |  |  |
| 45 | WPJG13 | 35 | HOUSE KEEPING | EP |  |  |  |
| 46 | WPMG22 | 44 | GOVERNMENT EMPLOYEE | P |  |  |  |
| 47 | WPMK075 | 20 | FACTORY | EP |  |  |  |
| 48 | WPMK074 | 36 | INSURANCE ADVISOR | EP |  |  |  |
| 49 | WPMK073 | 32 | SALES | P |  | DIABETIC |  |
| 50 | WPMG20 | 22 | FACTORY | P |  |  |  |
| 51 | WGKG90 | 19 | FACTORY | P |  |  |  |
| 52 | WPMK076 | 22 | SYSTEM WORK | EP |  |  |  |
| 53 | WPMK077 | 22 | RECEPTIONEST | EP |  |  |  |
| 54 | WGKK079 | 24 | FACTORY | P |  |  | Recuring |
| 55 | WPMK078 | 41 | GARMENTS | EP |  |  |  |
| 56 | WGKG88 | 23 | BOOK BINDING | P |  |  |  |
| 57 | WGKK080 | 32 | CARE TAKER | EP |  |  | Recurring |
| 58 | WGKK081 | 38 | HOTEL SUPERVISOR | P |  |  | Recurring |
| 59 | WGKG87 | 47 | ATTENDER | P | MDR |  |  |
| 60 | WPBG37 | 19 | SALES | EP |  |  |  |
| 61 | WGKG80 | 30 | FACTORY | P | MDR |  |  |
| 62 | WGRK082 | 27 | BANK | EP |  |  |  |
| 63 | WGGG91 | 30 | TEACHER | P |  |  |  |
| 64 | WDSG46 | 51 | PACKING SECTION | EP |  | DIABETIS |  |
| 65 | WDSK083 | 27 | ADVOCATE | EP |  |  |  |
| 66 | WDSK038 | 24 | COMPANY | EP |  |  |  |
| 67 | WPMG97 | 47 | HOUSE KEEPING | EP |  |  |  |
| 68 | WPMK024 | 18 | MIRRER FACTORY | P |  |  |  |
| 69 | WGSK066 | 17 | GARMENTS | P |  |  |  |
| 70 | WGSK084 | 32 | JEWELLERY SHOP | P | MDR |  | Recurring |
| 71 | WHEK085 | 33 | STUFF NURSE | EP |  |  |  |
| 72 | WHEK086 | 32 | OFFICER | P |  |  |  |
| 73 | WGSK065 | 20 | GARMENTS | EP |  |  |  |
| 74 | WGSK087 | 40 | GARMENTS | P |  |  | Recurring |
| 75 | WGSK088 | 19 | DOMESTIC HELP | EP |  |  |  |
| 76 | WGSK089 | 23 | SALES | P |  |  | Recurring |
| 77 | WGGG85 | 58 | IRON SHOP | P |  | DIABETES |  |
| 78 | WPJG16 | 21 | FACTORY | EP |  |  |  |
| 79 | WPJK090 | 44 | HOTEL | P |  |  |  |
| 80 | WPJK091 | 22 | STREET VENDOR | EP |  |  |  |
