# Supplementary material for: Intersections of informal work status, gender and tuberculosis diagnosis: Insights from a qualitative study from an Indian setting
Source: PLoS One. 2023 Jul 27;18(7):e0289137. doi: 10.1371/journal.pone.0289137 (PMC10374003; doi:10.1371/journal.pone.0289137)
Supplement: Attachment — Submitted filename: Plosone.docx [file pone.0289137.s003.docx]

The authors have done a good job on tuberculosis diagnosis, but this paper still needs improvement.

1. The abstract should always mention the rate of efficacy/efficiency percentage of the proposed method for the reader’s quick overview.
2. The abstract should at least have a line or two about the need for this work. This abstract has an intro and it straightaway deals with the proposed work.
3. Apart from giving the literature in paragraphs, better to add a table which precisely shows the proposed method, pros and cons.
4. At the end of the introduction section, there should be ‘Paper Structure / Paper Organization’.
5. The novelty of this paper is not clear. The difference between the present work and previous works should be highlighted. Add more of the issues and what is the significance of this research.
6. Before the paper's contributions, the author could precisely include the need to develop the proposed method.
7. The authors proposed architecture could be more precise, and a flow/sequence diagram with every step involved shall give it.
8. Analytical data representation should be more in-depth and comprehensive.
9. Among 22 references, hardly one is from 2023. This shows that the paper hasn’t considered more contemporary related works in the survey. Other than tuberculosis some common disease prediction should be mentioned in the literature. I suggest a few more papers to cite and refer.

<https://doi.org/10.1038/s41598-023-35922-x>

<https://dx.doi.org/10.2174/1574893618666230206121127>

DOI: 10.1504/IJDATS.2022.10053183

<https://doi.org/10.1111/exsy.13298>

10. A separate section for Limitations and future work in detail would give further ideas for the readers who wish to enhance your work.
