# Supplementary material for: Intersections of informal work status, gender and tuberculosis diagnosis: Insights from a qualitative study from an Indian setting
Source: PLoS One. 2023 Jul 27;18(7):e0289137. doi: 10.1371/journal.pone.0289137 (PMC10374003; doi:10.1371/journal.pone.0289137)
Supplement: Attachment — Submitted filename: Responses to reviewers.docx [file pone.0289137.s004.docx]

To

Editor

PLOS ONE

Sub: Rebuttal letter

Dear Sir/Madam

Please find below the responses and compliances to the comments of reviewers

| Comments | Response |
| --- | --- |
| The abstract should at least have a line or two about the need for this work. This abstract has an intro and it straightaway deals with the proposed work. | Added two sentences on the need of the work |
| The abstract should always mention the rate of efficacy/efficiency percentage of the proposed method for the reader’s quick overview. | The present paper is based on qualitative methods and the rate of efficacy/efficiency percentage of the proposed method cannot be computed |
| At the end of the introduction section, there should be ‘Paper Structure / Paper Organization’. | Included a paragraph on paper structure/organisation |
| The novelty of this paper is not clear. The difference between the present work and previous works should be highlighted. Add more of the issues and what is the significance of this research. | Added a paragraph to emphasize the novelty of the paper |
| Before the paper's contributions, the author could precisely include the need to develop the proposed method. | Included |
| The authors proposed architecture could be more precise, and a flow/sequence diagram with every step involved shall give it. | Included another diagram that shows the architecture related to development of themes from codes and code families (figure 1) |
| Analytical data representation should be more in-depth and comprehensive. | Added more data and analysis in the 'results' section |
| Among 22 references, hardly one is from 2023. This shows that the paper hasn’t considered more contemporary related works in the survey. Other than tuberculosis some common disease prediction should be mentioned in the literature | Cited 3 more relevant articles which are published in 2023 |
| A separate section for Limitations and future work in detail would give further ideas for the readers who wish to enhance your work. | Included |

Thank you

Sincerely

Sobin Geoge

5/07/2023
